# Detection and Control of Pregnancy Hypertension Using Self-Monitoring of Blood Pressure With Automated Telemonitoring: Cost Analyses of the BUMP Randomized Trials

**DOI:** 10.1161/HYPERTENSIONAHA.123.22059

**Published:** 2024-01-23

**Authors:** Helen E. Campbell, Lucy C. Chappell, Richard J. McManus, Katherine L. Tucker, Carole Crawford, Marcus Green, Oliver Rivero-Arias

**Affiliations:** 1 National Perinatal Epidemiology Unit, Nuffield Department of Population Health (H.E.C., O.R.-A.); 2 Nuffield Department of Primary Care Health Sciences. University of Oxford, United Kingdom (R.J.M., K.L.T., C.C.).; 3 Department of Women and Children’s Health, King’s College London, St Thomas’ Hospital, United Kingdom (L.C.C.).; 4 Action on Pre-eclampsia, Evesham, United Kingdom (M.G.).

**Keywords:** blood pressure, cost, hypertension, pregnancy, systolic blood pressure

## Abstract

**BACKGROUND:**

Pregnancy hypertension continues to cause maternal and perinatal morbidity. Two linked UK randomized trials showed adding self-monitoring of blood pressure (SMBP) with automated telemonitoring to usual antenatal care did not result in earlier detection or better control of pregnancy hypertension. This article reports the trials’ integrated cost analyses.

**METHODS:**

Two cost analyses. SMBP with usual care was compared with usual care alone in pregnant individuals at risk of hypertension (BUMP 1 trial [Blood Pressure Monitoring in High Risk Pregnancy to Improve the Detection and Monitoring of Hypertension], n=2441) and with hypertension (BUMP 2 trial, n=850). Clinical notes review identified participant-level antenatal, intrapartum, and postnatal care and these were costed. Comparisons between trial arms used means and 95% CIs. Within BUMP 2, chronic and gestational hypertension cohorts were analyzed separately. Telemonitoring system costs were reported separately.

**RESULTS:**

In BUMP 1, mean (SE) total costs with SMBP and with usual care were £7200 (£323) and £7063 (£245), respectively, mean difference (95% CI), £151 (−£633 to £936). For the BUMP 2 chronic hypertension cohort, corresponding figures were £13 384 (£1230), £12 614 (£1081), mean difference £323 (−£2904 to £3549) and for the gestational hypertension cohort were £11 456 (£901), £11 145 (£959), mean difference £41 (−£2486 to £2567). The per-person cost of telemonitoring was £6 in BUMP 1 and £29 in BUMP 2.

**CONCLUSIONS:**

SMBP was not associated with changes in the cost of health care contacts for individuals at risk of, or with, pregnancy hypertension. This is reassuring as SMBP in pregnancy is widely prevalent, particularly because of the COVID-19 pandemic.

**REGISTRATION:**

URL: https://www.clinicaltrials.gov; Unique identifier: NCT03334149.

**Novelty and Relevance**

**What Is New?**

In this study, data from the first adequately powered randomized controlled trials of blood pressure self-monitoring in pregnancy have shown it does not lead to an increase in the cost of health care contacts when added to usual antenatal care for pregnant individuals with or at risk of hypertension. A cost would however be associated with the purchase of the telemonitoring system.

**What Is Relevant?**

Each year, elevated blood pressure affects 18 million pregnancies worldwide and regular monitoring of blood pressure is necessary to prevent morbidity and mortality. In 2020, a rapid and unplanned rollout of self-monitoring of blood pressure among pregnant individuals with or at risk of hypertension was undertaken as a result of the COVID-19 pandemic. Work we previously published showed the act of self-monitoring by itself did not improve health outcomes but, reassuringly, it was both acceptable and empowering to pregnant persons. This study now provides evidence that self-monitoring of blood pressure during pregnancy is unlikely to lead to an increase in the cost of health care contacts.

**Clinical/ Pathophysiological Implications?**

Blood pressure self-monitoring is safe and does not increase health care contact costs when used in pregnant persons with or at risk of hypertension. Future research must now focus on the inclusion of interventions (medication and lifestyle-related) within the antenatal care pathway that can act to reduce elevated home blood pressure readings.

Annually, elevated blood pressure (BP) affects 18 million pregnancies worldwide; it is associated with preeclampsia and maternal and infant morbidity and mortality.^1,2^ In the United Kingdom and Ireland, deaths from preeclampsia are rare; however, a recent confidential enquiry into maternal deaths reported a death rate for 2018 to 2020 (0.38 per 100 000 maternities) that was 4× greater than seen during 2012 to 2014.^3^ Also noted was that for the foreseeable future, a significant number of pregnant individuals will be affected by preeclampsia and other hypertensive disorders. In the Unites States, the incidence of new-onset hypertensive disorders of pregnancy doubled between 2007 and 2019, with urban areas seeing an increase from 37.0 to 77.2 per 1000 live births.^4^

Increasing age, high body mass index, and the presence of comorbidities increase the risks of hypertension and preeclampsia during pregnancy. As BP can rise rapidly and between antenatal visits, regular BP measurement is considered important for individuals at risk of, and with, pregnancy hypertension. Self-monitoring of BP (SMBP), whereby individuals take their own BP readings outside of a clinical setting, has successfully been used to identify and guide the management of hypertension in nonpregnant individuals.^5–7^ A recent systematic review of economic analyses also suggested that SMBP is likely to be cost-effective.^8,9^

In the United Kingdom, the COVID-19 pandemic led SMBP in pregnancy to be rapidly adopted on a wide scale, with the National Health Service (NHS) providing some 16 000 home BP monitors in April 2020 alone.^10^ Until recently, however, the evidence base for SMBP in pregnancy was limited to small and mostly nonrandomized studies.^11–13^ The BUMP (Blood Pressure Monitoring in High Risk Pregnancy to Improve the Detection and Monitoring of Hypertension) program, was designed to develop and evaluate a SMBP intervention for use in pregnancy. The intervention comprised a validated automated monitor (Microlife WatchBP Home) for measuring BP at home at clinician-directed intervals, and a specially developed mobile phone-based telemonitoring system into which users manually entered their home BP readings.^14^ Elevated readings triggered an automated request for a further reading, which if still elevated, led to an individual receiving a further automated message to contact their local maternity unit. SMBP readings were not automatically transferred to an individual’s electronic patient records and monitored by clinicians, and no virtual consultations took place using the system. However, each week, hospitals received a summary of the SMBP readings for each of their participants. Health care professionals could also view an individual’s historic SMBP readings via their mobile phone display during consultations.

The BUMP program demonstrated this intervention was feasible and acceptable to pregnant individuals, who were empowered by self-monitoring, enabling them to be motivated and proactive in their own care.^13,15^ The program also included 2 linked randomized controlled trials assessing the effectiveness of the intervention.^16^ The BUMP 1 trial evaluated whether the intervention could lead to earlier detection of elevated clinic BP in individuals at risk of pregnancy hypertension, the BUMP 2 trial whether the intervention could lead to better BP control in individuals with pregnancy hypertension. Results showed the intervention did not lead to earlier hypertension detection (BUMP 1) or better control (BUMP 2).^17,18^ However, in BUMP 1, over half of the participants diagnosed with hypertension had elevated home BP readings, and insights from allied qualitative work suggested the lack of effect could in part have been a result of uncertainty around how SMBP readings should be incorporated within the clinical decision making process. Reassuringly, individuals self-monitoring experienced no additional harms, unexpected consequences or any detrimental impact upon their health-related quality of life.^17,18^

Integral to each BUMP trial was an economic evaluation, designed to quantify the costs and consequences of adding the SMBP intervention to usual antenatal care. With no differences in either trial’s clinical or participant-reported outcomes, we now report the cost analyses. If self-monitoring were to increase health care costs (eg, if any anxiety related to SMBP increased unscheduled health care contacts), it is questionable whether such additional costs (and indeed the widespread acceptance of SMBP in pregnancy seen because of the COVID-19 pandemic) could be justified purely on the basis of the motivation and empowerment it provides to pregnant individuals. However, if the cost impact was neutral or cost-saving, then the guidance around SMBP in pregnant individuals with or at risk of hypertension may be wholly different; we would have evidence to suggest that its widespread acceptance is unlikely to have led to an inefficient use of scarce health care resources.

## METHODS

The data that support the findings of this study are available from the authors at information.guardian@phc.ox.ac.uk upon reasonable request. The BUMP 1 and 2 trials have been described in detail elsewhere.^17,18^ Briefly, BUMP 1 randomized 2441 pregnant individuals at 16 to 24 weeks’ gestation and with a higher risk of preeclampsia, to SMBP 3× per week plus usual antenatal care (n=1223) or to usual antenatal care alone (n=1218). The primary outcome was time to first recording of hypertension (sustained BP of ≥140/90 mm Hg) measured by a health care professional, with the trial powered to detect a difference of 12 days. In BUMP 2850 pregnant individuals with hypertension were randomized to daily SMBP plus usual antenatal care (n=430) or to usual antenatal care alone (n=420). BUMP 2 included individuals with chronic hypertension (n=454), enrolled up to 37 weeks’ gestation, and with gestational hypertension (n=396), enrolled between 20 and 37 weeks’ gestation. The primary outcome was mean systolic BP measured by health care professionals between randomization and birth, with the trial powered to detect a 5 mm Hg difference. A linked design was used such that individuals initially recruited to BUMP 1 and who developed pregnancy hypertension, transitioned to BUMP 2, maintaining their original randomization with others recruited directly (particularly those with chronic hypertension) into BUMP 2. Both trials were approved by the research ethics committee (West Midlands-South Birmingham: ref 17/WM/0241). All participants gave written informed consent.

### Resource Use and Costs

Taking an NHS perspective, health care resource use data were extracted from each participant’s clinical records out to hospital discharge following delivery/end of the pregnancy. These data included antihypertensive medications prescribed, and counts of clinic visits, day/maternity assessment unit visits, and inpatient bed days during the antenatal period. Information on each participant’s delivery type, postdelivery inpatient length of stay, need for a blood transfusion, interhospital transfers, and pregnancies ending with a miscarriage or stillbirth were recorded. Inpatient days on the neonatal unit and ward and interhospital transfers were recorded for infants born to trial participants. For individuals and infants with prolonged hospital stays, data capture was censored at 2 months following delivery or the estimated date of delivery (whichever was longer).

Health care resource use data were costed using unit costs (2019/2020 UK £) from many established sources.^19–25^ These are shown in Table S1. The Supplemental File also provides details of the costing methods used. Discounting of costs was not necessary as the time horizon in each trial was limited by the length of pregnancy.

### SMBP Costs

For participants in the self-monitoring arms, costs associated with use of the home BP monitor were estimated (see costing methods in the Supplemental File).

The automated telemonitoring system was developed solely for the purposes of the BUMP trials, however, such systems can now be procured by the NHS from commercial providers. Using information from the NHS supplier’s portal, we estimated that the cost to the NHS of a hospital-based telemonitoring solution would be between £12 000 and £15 000 per hospital department per year. The mid-point of this cost range was multiplied by the estimated number of NHS Hospital Trusts providing antenatal care in England and Wales and a system cost per individual estimated by dividing this total cost by the number of pregnant persons in England and Wales either at risk of hypertension (BUMP 1) or already affected by hypertension (BUMP 2) during a given year. These system costs are reported alongside the cost-analysis results.

### Statistical Analysis

Analyses for BUMP 1 compared individuals in each arm of the trial. For BUMP 2, between arm comparisons were made separately for chronic hypertension and gestational hypertension cohorts, given evidence showing these groups could potentially vary in terms of their BP characteristics and the effect size of the intervention.^13,16^ For each trial arm, means, and SDs described resource use and costs for pregnant persons and infants with complete data. Mean differences and parametric 95% CIs were used when comparing between trial arms. CIs for participant costs were adjusted for study site and parity, and for infants, for site, parity, and twins. For the BUMP 2 gestational hypertension cohort, adjustments were also made for transitions into the trial from BUMP 1.

The amount of missing resource use data was small; total costs were not calculable for only 80/2437 (3.3%) participants in BUMP 1, and in BUMP 2, 17/454 (3.7%) participants with chronic hypertension and 7/396 (1.8%) with gestational hypertension. Multiple imputation was thus performed only for missing total cost estimates and appropriately adjusted means and SEs were reported.^26–29^ The Supplemental File provides details.

## RESULTS

Table 1 summarizes key baseline characteristics of individuals recruited to both trials, with full participant details reported elsewhere.^17,18^ Characteristics were well matched between arms in both trials. Tables 2 through 4 report mean resource use, costs and cost comparisons between arms for participants with complete data in BUMP 1 (Table 2), BUMP 2’s chronic hypertension cohort (Table 3), and BUMP 2’s gestational hypertension cohort (Table 4).

**Table 1.**
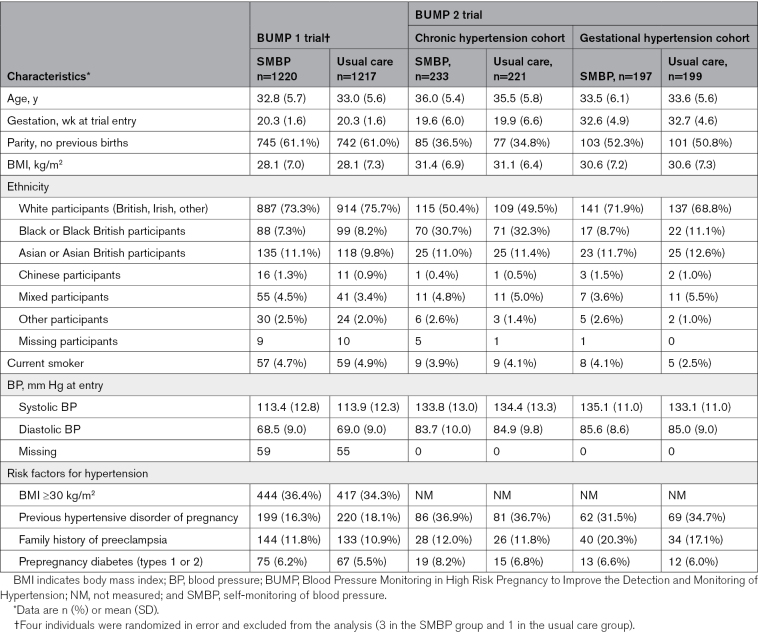
Key Baseline Characteristics of Participants in the BUMP 1 and BUMP 2 Trials

**Table 2.**
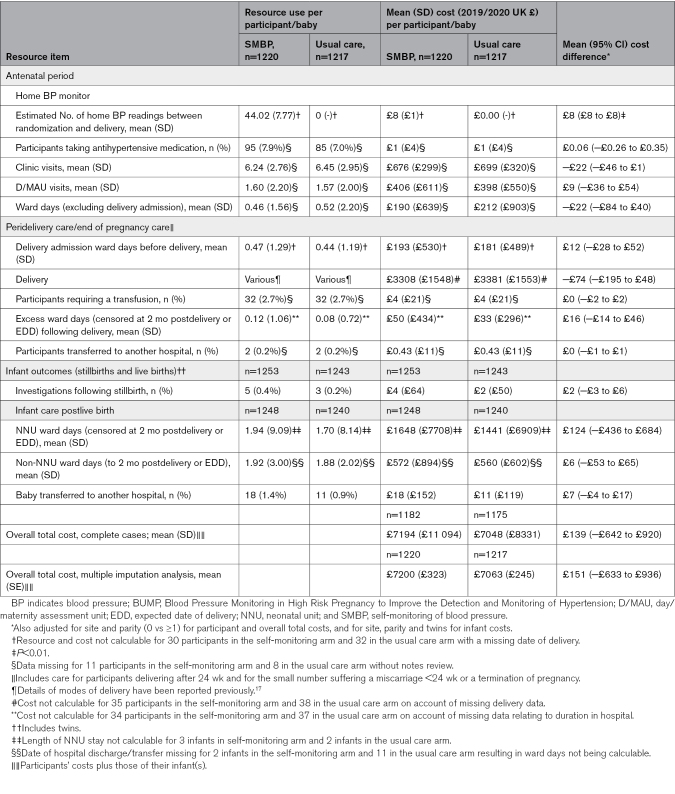
Resource Use, Costs, and Cost Differences Per Participant and Infant in BUMP 1

**Table 3.**
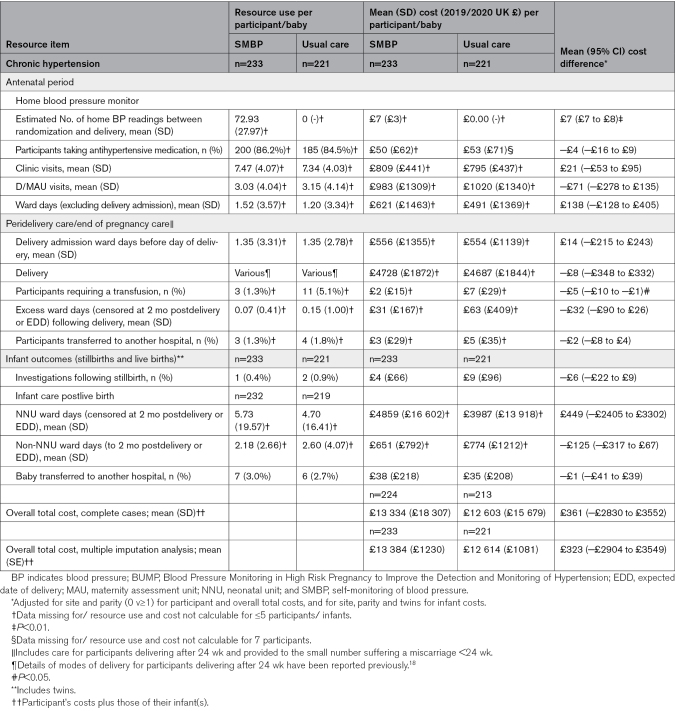
Resource Use, Costs, and Cost Differences Per Participant and Infant in BUMP 2: Chronic Hypertension Cohort Only

**Table 4.**
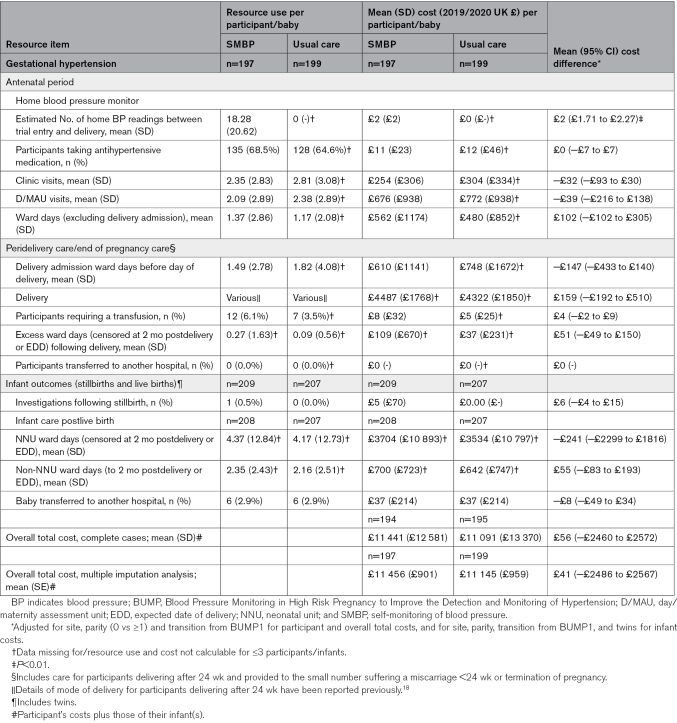
Resource Use, Costs, and Cost Differences Per Participant and Infant in BUMP 2: Gestational Hypertension Cohort Only

### BUMP 1

In BUMP 1, and apart from the small cost of the BP monitor, resource use during the antenatal period was similar between trial arms. There were also no differences in the care provided at the time of and immediately following delivery or in any infant costs. The mean total cost difference seen between the SMBP and usual care arms was nonsignificant at £139, 95% CI, −£642 to £920. The final row of Table 2 shows total cost figures changed little following multiple imputation.

### BUMP 2: Chronic Hypertension

Within the chronic hypertension cohort in BUMP 2, antenatal resource use, and costs were similar between the SMBP and usual care arms (Table 3). More maternal participants in the usual care arm required a transfusion; however, there were no other differences in the care individuals received at the time of and immediately following delivery. Overall, mean total costs did not differ between SMBP and usual care (mean cost difference with SMBP was £361, 95% CI, −£2830 to £3552). Again, there was little change to the total cost estimate following multiple imputation (final row of Table 3).

### BUMP 2: Gestational Hypertension

Individuals with gestational hypertension entered the BUMP 2 trial at a later gestation than those with chronic hypertension and thus had fewer antenatal contacts recorded (Table 4). Again, no cost differences were observed between trial arms during the antenatal period and at the time of delivery, and costs were also similar between infants born to individuals in each arm of the trial. The final row of Table 4 shows no significant difference in mean overall total costs (£41, 95% CI, −£2486 to £2567) between SMBP and usual care.

### Telemonitoring System Cost

Assuming a telemonitoring system cost of £13 500 per hospital department per year and a total of 138 NHS Hospital Trusts providing antenatal care in England and Wales, generated a total annual system cost of £1.86 million. Assuming that 50% of the 642 731 pregnancies resulting in a live birth or stillbirth in England and Wales in 2019, would have been in individuals at risk of pregnancy hypertension (BUMP 1), the resulting system cost per individual would be around £6. If implemented only for individuals with pregnancy hypertension (BUMP 2), then for the same total system cost, and assuming that 10% of pregnancies were affected by hypertension, the resulting cost per hypertensive individual would be £29.

## DISCUSSION

The cost analyses presented here were part of the first adequately powered randomized controlled trials of SMBP in pregnancy hypertension. The trials showed that although the SMBP intervention did not lead to earlier detection or better control of pregnancy hypertension, it did not seem to be harmful. Allied work also showed SMBP generated feelings of motivation and empowerment in pregnant individuals. The analyses reported in this article have further shown that the addition of the SMBP intervention to usual care did not increase antenatal contacts or indeed total health care costs for pregnant individuals at risk of, or with, hypertension. With questions previously raised around the psychological impact of SMBP and whether it induces feelings of anxiety that may lead individuals to seek additional unscheduled health care consultations, the results reported here are reassuring, and accord with other findings from the BUMP program suggesting the addition of SMBP to routine antenatal care appears to be both safe and acceptable.^30^ Given the increased reliance on SMBP initially compelled upon the maternity community following the advent of the COVID-19 pandemic in 2020 and now being incorporated into usual care, these findings are even more pertinent.^10^

Although we reported the per individual costs of the telemonitoring system itself (£6 for BUMP 1 and £29 for BUMP 2) alongside the cost-analysis results, had we included them in the mean total cost estimates shown in Tables 2, 3, and 4, the overall mean cost differences between the trial arms would have remained nonsignificant. Nevertheless, it is important to consider the costs of telemonitoring system implementation at a national level, particularly as the total population of individuals at whom the system would be aimed, is large. In this instance, and if telemonitoring systems were to be purchased for hospitals providing antenatal care in England and Wales, the total cost is likely to be around £1.9 million annually. For policy makers and planners considering whether to formally adopt a new intervention, having such information on the potential costs of system implementation is important.

The analyses presented here used a common methodology to generate descriptions and associated costs of antenatal, perinatal, and postnatal care provided to 3 different and clinically distinct groups of individuals in the field of pregnancy hypertension. In addition to the comparative analyses reported for each group, the work also facilitates a consideration of how costs vary between groups. Looking at the at risk (Table 2) and chronic hypertension (Table 3) groups, for example shows total health care costs for the latter to be almost twice those of the former; the cost difference driven by greater numbers of antenatal contacts (outpatient and inpatient care), more costly delivery types, and the need for more neonatal unit care for infants following birth. For clinicians, economists and policy makers considering new care pathways containing interventions which act upon SMBP readings (eg, self-titration of antihypertensive medication), these data provide an insight into which individuals may have the greater capacity to benefit, and thus for whom such interventions are likely to be cost-effective.

Increasing interest in SMBP for pregnancy hypertension has seen many economic analyses published.^31–33^ However, in contrast to the BUMP work where SMBP was implemented in addition to routine antenatal care, these other studies have, in the main, evaluated SMBP as a substitute for some face-to-face antenatal consultations. In the Netherlands, for example, van den Heuvel et al^31^ used a case-control design with 230 individuals to evaluate a SMBP intervention which was coupled with a reduction in routine antenatal contacts for individuals predominantly at risk of pregnancy hypertension. Similarly, in the United Kingdom, Xydopoulos et al^32^ undertook a case-control study of 166 individuals with pregnancy hypertension and compared SMBP plus individualized schedules of less frequent midwife contacts, with routine antenatal care alone. Both studies reported that the new care pathways were not associated with any adverse pregnancy outcomes and brought about reductions in antenatal contacts and health care costs. Such findings are intuitive if submitted SMBP readings are used as a substitute for certain face-to-face antenatal visits.

The work presented here has many strengths. First, the findings are based upon data collected during the first adequately powered, socio-demographically representative randomized controlled trials of SMBP in pregnancy hypertension and thus are likely to be generalizable with a low risk of bias.^17,18^ Previous economic analyses in the area utilized case-control designs with small numbers of participants and observed imbalances in some patient characteristics between study arms.^31–33^ Second, study cost estimates are based upon data extracted from medical records with high levels of completeness. Third, and given that the proportion of pregnant individuals affected by pregnancy hypertension will remain similar, this work has generated reliable cost estimates which can be utilized in future economic evaluations in the area.

This study is not without its limitations. It was necessary to make assumptions when costing. For example, data were not available on the types of professionals that participants saw at antenatal clinics (nurses, midwives, or obstetricians), or on the levels of care (intensive care, high dependency care, special care) provided to infants in the neonatal unit (see Supplemental File). When costing this care we used weighted average unit costs, in effect allowing only the number of contacts/duration of stay to vary between trial arms and not the intensity of the care provided. Had the BUMP trials demonstrated differences in primary and secondary outcomes between SMBP and usual care, then, it would have been prudent to conduct extensive sensitivity analyses around these assumptions. However, and given the lack of effect across end points in BUMP 1 and BUMP 2, such analyses are unwarranted.

As called for by the BUMP trial publications, research should now focus on the development and evaluation of interventions within the care pathway that act upon elevated SMBP readings. Examples may include the automated transfer of readings to electronic patient records, self-managed titration of antihypertensive medication, or lifestyle counseling. Of course, the coupling of such interventions with SMBP will have resource implications. For example additional clinical/nursing time would be required to review and act upon raised SMBP readings transferred to electronic patient records. On the contrary, increased monitoring frequency and more timely responses to rises in SMBP, may permit amendments to be made to the intensity of the routine care monitoring schedules of individuals. Alongside assessments of clinical benefit, future research would need to carefully examine the net cost impact of such coupled interventions.

## CONCLUSIONS

This work has shown that SMBP in pregnancy hypertension, as implemented in the BUMP trials, does not increase health care costs. Implementation at a national level would however require the purchase of a telemonitoring system. Although SMBP did not lead to earlier diagnosis or better control of BP, the findings reported here, when coupled with the data showing SMBP is both safe and acceptable to pregnant individuals, are reassuring, especially given the rapid and widespread adoption of SMBP in pregnancy seen following the COVID-19 pandemic.

## PERSPECTIVES

BP can increase rapidly during pregnancy and can lead to morbidity and mortality for the pregnant individual and the infant. Regular BP measurement is necessary for individuals both at risk of, and with, pregnancy hypertension. The BUMP program developed a SMBP intervention for use in this patient group. Evaluation showed the intervention did not lead to earlier detection or better control of hypertension with uncertainty around how to integrate elevated home BP readings within the clinical decision making process a possible contributing factor.^10^ Importantly, analyses showed that SMBP was safe, acceptable, and empowering for pregnant individuals and this article presents a further component of the work demonstrating that SMBP did not increase the costs of health care contacts. Costs would however be incurred by the purchase of the telemonitoring system. Such findings are reassuring given the forced and unplanned roll out of SMBP in pregnancy during the COVID-19 pandemic. They also imply that SMBP offers an acceptable means of measuring BP away from the clinical setting and that work should now focus upon identifying and evaluating interventions that can be incorporated within the clinical care pathway to act upon elevated home BP readings.

## ARTICLE INFORMATION

### Acknowledgments

The authors are grateful to the participants who took part in the Blood Pressure Monitoring in High Risk Pregnancy to Improve the Detection and Monitoring of Hypertension program of work, along with the site research midwives and physicians. The authors would also like to extend thanks to the independent trial steering and data monitoring committees, and to the study’s patient and public involvement representatives. The authors are grateful to Lucy Curtin, for administrative support, and Lucy Abel, who did preparatory health economic work on the project.

### Sources of Funding

This work was funded from a National Institute for Health and Care Research (NIHR) program grant for applied research (RP-PG-1209-10051) and NIHR professorships awarded to R.J. McManus (NIHR-RP-R2-12-015) and L.C. Chappell (NIHR-RP-2014-05-019). R.J. McManus and K.L. Tucker received funding from the NIHR Collaboration for Leadership in Applied Health Research now recommissioned as NIHR Applied Research Collaboration Oxford and Thames Valley. R.J. McManus and L.C. Chappell are NIHR Senior Investigators. Service support costs were administered through the NIHR Clinical Research Network.

### Disclosures

L.C. Chappell reported serving as Chief Scientific Adviser to the UK Department of Health and Social Care and Chief Executive Officer for the National Institute for Health and Care Research (NIHR) since August 2021. The Blood Pressure Monitoring in High Risk Pregnancy to Improve the Detection and Monitoring of Hypertension telemonitoring intervention was developed into a commercial product in collaboration with Sensyne Health and licensed by Oxford University and Sensyne Health for free during the pandemic. The University has received fees subsequently from Sensyne following the conduct of the study. R.J. McManus reported receiving fees from Sensyne following the conduct of the study and nonfinancial support from Omron (Omron licensed and paid consultancy to the University of Oxford with regard to a telemonitoring intervention developed with his help, and previously supplied blood pressure monitors for the TASMINH4 study). R.J. McManus also reported occasional travel and accommodation for speaking at conferences (any honoraria are paid to his institution). The other authors report no conflicts.

### Supplemental Material

Costing Methodology

Multiple Imputation Methodology

Table S1

## Supplementary Material
