# Supplementary material for: Detection and Control of Pregnancy Hypertension Using Self-Monitoring of Blood Pressure With Automated Telemonitoring: Cost Analyses of the BUMP Randomized Trials
Source: Hypertension. 2024 Jan 23;81(4):887–96. doi: 10.1161/HYPERTENSIONAHA.123.22059 (PMC10956677; doi:10.1161/HYPERTENSIONAHA.123.22059)
Supplement: Supplementary file 1 [file hyp-81-887-s001.docx]

**Supplementary Materials**

**Detection and control of pregnancy hypertension using self-monitoring of blood pressure with automated telemonitoring: cost analyses of the BUMP randomised trials.**

Helen E. Campbell DPhil^1^; Lucy C. Chappell, MB BChir, PhD^2^; Richard J. McManus, MBBS, PhD^3^; Katherine L. Tucker, PhD^3^; Carole Crawford, MSc^3^; Marcus Green, BA(Hons)^4^; Oliver Rivero-Arias, DPhil^1^

^1^National Perinatal Epidemiology Unit, Nuffield Department of Population Health, University of Oxford, Oxford, UK.

^2^Department of Women and Children’s Health, King’s College London, St Thomas’ Hospital, London, UK.

^3^Nuffield Department of Primary Care Health Sciences, University of Oxford, Oxford, UK.

^4^Action on Pre-eclampsia, The Stables, 80B High Street, Evesham, UK.

**Corresponding Author:**

Associate Professor Oliver Rivero-Arias, National Perinatal Epidemiology Unit, Nuffield Department of Population Health, University of Oxford, Oxford, UK. OX3 7LF. Email oliver.rivero@npeu.ox.ac.uk

**Costing methodology used in both trials**

Table S1 lists the unit costs used to value the resource use data collected during the trial.

**Additional information on the costing of antenatal care**

*Antihypertensive medication*

At 20 weeks’ gestation, 30 weeks’ gestation, and in the week prior to delivery or the end of the pregnancy, data were recorded on the type, dose, method of administration and frequency of antihypertensive medications prescribed to individuals. In BUMP 2, if the start date of an individual’s medication was prior to entry into the trial, medication was costed only from the day they entered the trial. When a medication at one time point was not recorded at the following time point, the individual was assumed to have ceased taking it mid-way between time points.

The date of dose changes to medications was not always recorded (the CRF requested only the date the medication was first prescribed). This occurred in a small number of participants taking the same medication at 30 weeks and at the end of pregnancy. For these individuals, the dose change was assumed to have taken place at the start of the week (7 days) prior to delivery, which was the last data recording point prior to delivery. Medications were costed up to delivery or the end of pregnancy using relevant unit costs taken primarily from the August 2020 NHS Electronic Drug Tariff (table S1).[1]

*Clinic Visits / Maternity Assessment Unit visits*

The dates on which an individual’s blood pressure was measured at a clinic or a day / maternity assessment unit (D/MAU) were used to make a count of the number of visits for each type of contact post-trial entry and prior to delivery. Duplicate dates were excluded (when >1 blood pressure measurement was taken at the same contact). Data were not available on the type of health professional an individual saw at each clinic visit and so a weighted average of unit costs across consultant and non-consultant led follow-up clinics in obstetric and midwifery specialities was estimated (table S1).[2, 3] When costing D/MAU visits, a distinction was made between individuals with and without hypertension. For the former, visits were costed at an intermediate care level as indicated by the NHS maternity care pathway, and for the latter, at a standard level (table S1) [4].

*Ward days prior to delivery*

The dates on which a participant’s blood pressure was measured on an inpatient ward were used to make a count of the number inpatient bed days post-trial entry and prior to delivery. Information on the level of care provided when an individual was an inpatient was not available. It was assumed participants would most likely be being monitored during these admissions and so they were costed using an excess bed day ward cost of £410 taken from the 2019/2020 NHS maternity pathway of the National Tariff Payment System: non-mandatory currencies and prices (table S1).[4]

**Additional information on the costing of peri-delivery care / end of pregnancy care**

*Delivery*

As the NHS National Cost Collection provides costs for each delivery type according to an individual’s level of co-morbidities / complications, to facilitate a more accurate costing of delivery, an individual’s level of co-morbidity / complications was first set to 0 or 1 to reflect the absence or presence of hypertension. Further co-morbidities were counted based upon the following baseline data (presence of autoimmune disease, diabetes, chronic kidney disease, a BMI>=30kg/m^2^).[2, 3] Participants with a post-delivery length of stay of 1 day or less were assigned the relevant short stay cost for their delivery type and co-morbidity level, whereas those remaining in hospital for longer were assigned the appropriate long stay cost. For these individuals, the trim point associated with their delivery type and co-morbidity level was identified, and the number of days in hospital beyond this trim point were costed using the excess bed day ward cost of £410 taken from the 2019/2020 maternity pathway of the National Tariff Payment System: non-mandatory currencies and prices (table S1).[4] In-hospital stays were censored at two months following delivery or the estimated date of delivery (whichever was longer).

When a twin pregnancy was recorded and the mode of delivery was the same for both babies, only a single delivery cost was assigned. If the babies were delivered in different ways (for example first baby by spontaneous vaginal delivery and the second baby by emergency Caesarean section), the cost of the most resource intensive delivery mode was assigned. For individuals undergoing a pregnancy termination or suffering a miscarriage <24 weeks’ gestation and being admitted to hospital, the appropriate unit costs from the NHS cost collection were assigned (table S1).[2, 3] For participants suffering a stillbirth, in addition to the cost of delivering the baby, additional costs were included for a post-mortem and the suite of core investigations routinely conducted (table S1).[5]

*Transfusion*

Data were recorded on whether a participant required a transfusion of any blood product during their hospital admission for delivery, but not on the product type or the number of units given. For the analysis, the cost of a single unit of red blood cells was assigned to individuals coded as receiving a transfusion (table S1).[6]

*Transfers to another hospital*

A small number of individuals were transferred to another hospital following delivery. Interrogation of the data suggested these participants were being transferred from tertiary hospitals in the absence of any complications and were unlikely to have been critical care transfers. They were therefore costed using the standard ambulance transfer cost shown in table S1.[2, 3]

**Additional information on the costing of infant care**

Admissions to a neonatal unit (NNU) and length of stay were recorded, however data were not available on the level of care (intensive care, high dependency, special care) received. Days on the NNU unit were therefore costed using a weighted average of the *per diem* costs of neonatal intensive care, high dependency care, and special care taken from the NHS National Cost Collection (table S1).[2, 3] Non-NNU days were also recorded and costed assuming a normal level of care (table S1).[2, 3] As for maternal inpatient stay following delivery, hospital stays for babies were censored at two months following delivery or estimated date of delivery (whichever was longer). When babies were transferred to other hospitals for further care, these transfers were costed using the unit cost shown in table S1.[2, 3]

**Additional information on the costing of the home BP monitor**

Using the purchase price of a home BP device (see table S1), an assumed device lifespan of two years, and a discount rate of 3%, an equivalent annual cost for the device was estimated. Within each trial, this cost was divided through by annual expected days of usage based upon the trial’s SMBP protocol and average adherence levels. The resulting cost per BP measurement day was then used to multiply the expected number of measurements for each participant based on their time in the trial and the protocol being followed.

**Multiple Imputation methodology**

Multiple imputation was performed only for missing total cost estimates, in BUMP 1 and separately for the chronic hypertension and gestational hypertension cohorts in BUMP 2.[7] For each imputation exercise, a model was constructed with covariates that included participant’s baseline characteristics at trial entry and their health related quality of life scores as measured using the EuroQol EQ-5D-5L questionnaire at baseline, 30 weeks and 12 weeks postnatally.[8] For the gestational hypertension cohort in BUMP 2, transition from BUMP 1 was also included as an additional covariate. Predictive mean matching, estimating 50 imputations, was implemented separately by trial allocation. Means and standard errors (SE) for total costs in each trial arm were estimated using Rubin’s rule to combine estimates across imputed datasets, with the use of linear models to make adjustments for study site, parity, potential EQ-5D-5L index baseline imbalances, and, for the gestational hypertension cohort in BUMP 2, transitions from BUMP 1.[9] Mean differences and 95% CIs were used when comparing total costs between trial arms.

**References**

1. NHS Business Services Authority and NHS Prescription Services. Electronic Drug Tariff [Internet]. [cited 15th August 2020]. Available from: <https://www.nhsbsa.nhs.uk/pharmacies-gp-practices-and-appliance-contractors/drug-tariff>.

2. National Health Service England. National Schedule of NHS Costs 2018/19 [Internet]. [cited 17th October 2020]. Available from: <https://www.england.nhs.uk/publication/2018-19-national-cost-collection-data-publication/>.

3. Curtis L, Burns A. Unit Costs of Health and Social Care 2020. Canterbury: Personal Social Services Research Unit, University of Kent; 2020.

4. NHS Improvement and NHS England. 2019/2020 National Tariff Payment System: non-mandatory currency and prices. [Internet]. [cited 17th October 2020]. Available from: <https://www.england.nhs.uk/publication/past-national-tariffs-documents-and-policies/>.

5. Campbell HE, Kurinczuk JJ, Heazell A, Leal J, Rivero-Arias O. Healthcare and wider societal implications of stillbirth: a population-based cost-of-illness study. BJOG : an international journal of obstetrics and gynaecology. 2018;125(2):108-17.

6. NHS Blood and Transplant Board. Pricing Proposals for 2019-20 and 2020-21. NHS Blood and Transplant. [Available from: <https://nhsbtdbe.blob.core.windows.net/umbraco-assets-corp/16611/ncg-pricing-proposals-board-paper-19_62.pdf>.

7. Faria R, Gomes M, Epstein D, White IR. A guide to handling missing data in cost-effectiveness analysis conducted within randomised controlled trials. PharmacoEconomics. 2014;32(12):1157-70.

8. Herdman M, Gudex C, Lloyd A, Janssen M, Kind P, Parkin D, et al. Development and preliminary testing of the new five-level version of EQ-5D (EQ-5D-5L). Quality of life research : an international journal of quality of life aspects of treatment, care and rehabilitation. 2011;20(10):1727-36.

9. Little RJ, Rubin DB. Statistical Analysis with Missing Data. Hoboken, NJ, : Wiley; 2002.

10. British Medical Association and Royal Pharmaceutical Society. British National Formulary [Internet]. [cited 15th August 2020]. Available from: <https://www.bnf.org/products/bnf-online/>.

**Table S1 Unit costs (2019/20 UK£) used to value healthcare resource use and contacts in the BUMP 1 and BUMP 2 trials**

| **Resource Use Item** | **Unit Cost** | **Source** |
| --- | --- | --- |
| ***Antenatal resource use*** |  |  |
| *Validated automated blood pressure monitor* | £42.50 | Personal communication, trial personnel |
| *Anti-hypertensive medication* |  |  |
| Labetalol 100mg/20mL injection ampoule £119.65 for 5 | £23.93 | NHS Electronic Drug Tariff, August 2020[1] |
| Labetalol 50mg tablets £3.79 for 56 | £0.07 | NHS Electronic Drug Tariff, August 2020[1] |
| Labetalol 100mg tablets £7.24 for 56 | £0.13 | NHS Electronic Drug Tariff, August 2020[1] |
| Labetalol 200mg tablets £10.20 for 56 | £0.18 | NHS Electronic Drug Tariff, August 2020[1] |
| Labetalol 400mg tablets £19.64 for 56 | £0.35 | NHS Electronic Drug Tariff, August 2020[1] |
| Nifedipine 10mg modified release capsules £3.90 for 60 | £0.07 | NHS Electronic Drug Tariff, August 2020[1] |
| Nifedipine 20mg modified release capsules £5.41 for 60 | £0.09 | NHS Electronic Drug Tariff, August 2020[1] |
| Nifedipine 30mg modified release capsules £4.89 for 28 | £0.17 | NHS Electronic Drug Tariff, August 2020[1] |
| Nifedipine 40mg modified release tablets £14.40 for 30 | £0.48 | NHS Electronic Drug Tariff, August 2020[1] |
| Nifedipine 60mg modified release capsules £7.34 for 28 | £0.26 | NHS Electronic Drug Tariff, August 2020[1] |
| Methyldopa 125mg tablets £103.21 for 56 | £1.84 | NHS Electronic Drug Tariff, August 2020[1] |
| Methyldopa 250mg tablets £23.11 for 56 | £0.41 | NHS Electronic Drug Tariff, August 2020[1] |
| Methyldopa 500mg tablets £17.92 for 56 | £0.32 | NHS Electronic Drug Tariff, August 2020[1] |
| Hydralazine 25mg tablets £6.25 for 56 | £0.11 | NHS Electronic Drug Tariff, August 2020[1] |
| Hydralazine 50mg tablets £8.66 for 56 | £0.15 | NHS Electronic Drug Tariff, August 2020[1] |
| Hydralazine 20mg powder for solution for injection £74.17 for 5 | £14.83 | British National Formulary[10] |
| Amlodipine 5mg tablets 91p for 28 | £0.03 | NHS Electronic Drug Tariff, August 2020[1] |
| Amlodipine 10mg tablets 96p for 28 | £0.03 | NHS Electronic Drug Tariff, August 2020[1] |
| Atenolol 25mg tablets 76p for 28 | £0.03 | NHS Electronic Drug Tariff, August 2020[1] |
| Atenolol 50mg tablets 78p for 28 | £0.03 | NHS Electronic Drug Tariff, August 2020[1] |
| Propranolol 10mg tablets at £1.84 for 28 | £0.07 | NHS Electronic Drug Tariff, August 2020[1] |
| Propranolol 40mg tablets at £1.77 for 28 | £0.06 | NHS Electronic Drug Tariff, August 2020[1] |
| Propranolol 80mg tablets at £2.72 for 56 | £0.05 | NHS Electronic Drug Tariff, August 2020[1] |
| Doxazosin 1mg tablets at £1.00 for 28 | £0.04 | NHS Electronic Drug Tariff, August 2020[1] |
| Doxazosin 2mg tablets at £1.04 for 28 | £0.04 | NHS Electronic Drug Tariff, August 2020[1] |
| Doxazosin 4mg tablets at £1.20 for 28 | £0.04 | NHS Electronic Drug Tariff, August 2020[1] |
| Doxazosin 8mg tablets at £6.46 for 28 | £0.23 | NHS Electronic Drug Tariff, August 2020[1] |
| Metorpolol 50mg tablets at £2.85 for 28 | £0.10 | NHS Electronic Drug Tariff, August 2020[1] |
| Metoprolol 100mg tablets at £2.94 for 28 | £0.11 | NHS Electronic Drug Tariff, August 2020[1] |
| Amiloride 5mg tablets at £33.09 for 28 | £1.18 | NHS Electronic Drug Tariff, August 2020[1] |
| Ramipril 5mg tablets at £1.55 for 28 | £0.06 | NHS Electronic Drug Tariff, August 2020[1] |
| *Ante-natal visits* |  |  |
| MAU attendance – standard pathway* | £210.00 | 2019/2020 National Tariff Payment System: non-mandatory maternity prices - Non-mandatory antenatal assessment visit[4] |
| MAU attendance – intermediate pathway† | £324.00 | 2019/2020 National Tariff Payment System: non-mandatory maternity prices - Non-mandatory antenatal assessment visit[4] |
| Antenatal clinic attendance‡ | £108.32 | Weighted average of face to face consultant and non-consultant led follow-up outpatient clinic visits across obstetric and midwifery specialties, National Schedule of NHS Costs 2018-19 inflated using Curtis LA & Burns A. Unit Costs of Health and Social Care 2020[2, 3] |
| *Inpatient care* |  |  |
| Maternal inpatient bed day§ | £410.00 | Excess bed day cost taken from 2019/2020 National Tariff Payment System: non-mandatory maternity prices[4] |
| *In-hospital delivery* |  |  |
| Spontaneous vaginal delivery, CC score 0, without induction, LOS 1 night | £1,637.64 | HRG Code NZ30C short stay. National Schedule of NHS Costs 2018-19 inflated using Curtis LA & Burns A. Unit Costs of Health and Social Care 2020[2, 3] |
| Spontaneous vaginal delivery, CC score 1, without induction, LOS 1 night | £1,696.61 | HRG Code NZ30B short stay. National Schedule of NHS Costs 2018-19 inflated using Curtis LA & Burns A. Unit Costs of Health and Social Care 2020[2, 3] |
| Spontaneous vaginal delivery, CC score 2+, without induction, LOS 1 night | £1,745.22 | HRG Code NZ30A short stay. National Schedule of NHS Costs 2018-19 inflated using Curtis LA & Burns A. Unit Costs of Health and Social Care 2020[2, 3] |
| Spontaneous vaginal delivery, CC score 0, without induction, LOS >1 and <=5 | £3,027.93 | HRG Code NZ30C non-elective long stay. National Schedule of NHS Costs 2018-19 inflated using Curtis LA & Burns A. Unit Costs of Health and Social Care 2020[2, 3] |
| Spontaneous vaginal delivery, CC score 1, without induction, LOS >1 and <=5 | £3,152.33 | HRG Code NZ30B non-elective long stay. National Schedule of NHS Costs 2018-19 inflated using Curtis LA & Burns A. Unit Costs of Health and Social Care 2020[2, 3] |
| Spontaneous vaginal delivery, CC score 2+, without induction, LOS >1 and <=5 | £3,354.28 | HRG Code NZ30A non-elective long stay. National Schedule of NHS Costs 2018-19 inflated using Curtis LA & Burns A. Unit Costs of Health and Social Care 2020[2, 3] |
| Spontaneous vaginal delivery, CC score of 0, with induction, LOS 1 night | £1,901.04 | Weighted average of short stay codes NZ31C, NZ32C, NZ33C, NZ34C. National Schedule of NHS Costs 2018-19 inflated using Curtis LA & Burns A. Unit Costs of Health and Social Care 2020[2, 3] |
| Spontaneous vaginal delivery, CC score of 1, with induction, LOS 1 night | £2,014.02 | Weighted average of short stay codes NZ31B, NZ32B, NZ33B, NZ34B. National Schedule of NHS Costs 2018-19 inflated using Curtis LA & Burns A. Unit Costs of Health and Social Care 2020[2, 3] |
| Spontaneous vaginal delivery, CC score of 2+, with induction, LOS 1 night | £2,065.82 | Weighted average of short stay codes NZ31A, NZ32A, NZ33A, NZ34A. National Schedule of NHS Costs 2018-19 inflated using Curtis LA & Burns A. Unit Costs of Health and Social Care 2020[2, 3] |
| Spontaneous vaginal delivery, CC score 0, with induction, LOS >1 and <=5 | £3,430.90 | Weighted average of non-elective long stay codes NZ31C, NZ32C, NZ33C, NZ34C. National Schedule of NHS Costs 2018-19 inflated using Curtis LA & Burns A. Unit Costs of Health and Social Care 2020[2, 3] |
| Spontaneous vaginal delivery, CC score 1, with induction, LOS >1 and <=6 | £3,710.81 | Weighted average of non-elective long stay codes NZ31B, NZ32B, NZ33B, NZ34B. National Schedule of NHS Costs 2018-19 inflated using Curtis LA & Burns A. Unit Costs of Health and Social Care 2020[2, 3] |
| Spontaneous vaginal delivery, CC score 2+, with induction, LOS >1 and <=6 | £4,087.29 | Weighted average of non-elective long stay codes NZ31A, NZ32A, NZ33A, NZ34A. National Schedule of NHS Costs 2018-19 inflated using Curtis LA & Burns A. Unit Costs of Health and Social Care 2020[2, 3] |
| Assisted vaginal delivery, CC score 0, without induction, LOS 1 night | £1,701.95 | HRG Code NZ40C short stay. National Schedule of NHS Costs 2018-19 inflated using Curtis LA & Burns A. Unit Costs of Health and Social Care 2020[2, 3] |
| Assisted vaginal delivery, CC score 1, without induction, LOS 1 night | £1,831.62 | HRG Code NZ40B short stay. National Schedule of NHS Costs 2018-19 inflated using Curtis LA & Burns A. Unit Costs of Health and Social Care 2020[2, 3] |
| Assisted vaginal delivery, CC score 2+, without induction, LOS 1 night | £1,827.75 | HRG Code NZ40A short stay. National Schedule of NHS Costs 2018-19 inflated using Curtis LA & Burns A. Unit Costs of Health and Social Care 2020[2, 3] |
| Assisted vaginal delivery, CC score 0, without induction, LOS >1 and <=5 | £3,189.53 | HRG Code NZ40C non-elective long stay. National Schedule of NHS Costs 2018-19 inflated using Curtis LA & Burns A. Unit Costs of Health and Social Care 2020[2, 3] |
| Assisted vaginal delivery, CC score 1, without induction, LOS >1 and <=6 | £3,502.71 | HRG Code NZ40B non-elective long stay. National Schedule of NHS Costs 2018-19 inflated using Curtis LA & Burns A. Unit Costs of Health and Social Care 2020[2, 3] |
| Assisted vaginal delivery, CC score 2+, without induction, LOS >1 and <=6 | £3,998.84 | HRG Code NZ40A non-elective long stay. National Schedule of NHS Costs 2018-19 inflated using Curtis LA & Burns A. Unit Costs of Health and Social Care 2020[2, 3] |
| Assisted vaginal delivery, CC score 0, with induction, LOS 1 night | £2,206.43 | Weighted average of short stay codes NZ41C, NZ42C, NZ4C, NZ44C. National Schedule of NHS Costs 2018-19 inflated using Curtis LA & Burns A. Unit Costs of Health and Social Care 2020[2, 3] |
| Assisted vaginal delivery, CC score 1, with induction, LOS 1 night | £2,346.59 | Weighted average of short stay codes NZ41B, NZ42B, NZ43B, NZ44B. National Schedule of NHS Costs 2018-19 inflated using Curtis LA & Burns A. Unit Costs of Health and Social Care 2020[2, 3] |
| Assisted vaginal delivery, CC score 2+, with induction, LOS 1 night | £2,408.71 | Weighted average of short stay codes NZ41A, NZ42A, NZ43A, NZ44A. National Schedule of NHS Costs 2018-19 inflated using Curtis LA & Burns A. Unit Costs of Health and Social Care 2020[2, 3] |
| Assisted vaginal delivery, CC score 0, with induction, LOS >1 and <=6 | £4,039.99 | Weighted average of non-elective long stay codes NZ41C, NZ42C, NZ4C, NZ44C. National Schedule of NHS Costs 2018-19 inflated using Curtis LA & Burns A. Unit Costs of Health and Social Care 2020[2, 3] |
| Assisted vaginal delivery, CC score 1, with induction, LOS >1 and <=7 | £4,498.94 | Weighted average of non-elective long stay codes NZ41B, NZ42B, NZ43B, NZ44B. National Schedule of NHS Costs 2018-19 inflated using Curtis LA & Burns A. Unit Costs of Health and Social Care 2020[2, 3] |
| Assisted vaginal delivery, CC score 2+, with induction, LOS >1 and <=10 | £5,096.65 | Weighted average of non-elective stay codes NZ41A, NZ42A, NZ43A, NZ44A. National Schedule of NHS Costs 2018-19 inflated using Curtis LA & Burns A. Unit Costs of Health and Social Care 2020[2, 3] |
| Emergency caesarean section with CC Score 0-1, LOS 1 night | £2,822.85 | HRG Code NZ51C short stay. National Schedule of NHS Costs 2018-19 inflated using Curtis LA & Burns A. Unit Costs of Health and Social Care 2020[2, 3] |
| Emergency caesarean section with CC Score 2-3, LOS 1 night | £2,973.02 | HRG Code NZ51B short stay. National Schedule of NHS Costs 2018-19 inflated using Curtis LA & Burns A. Unit Costs of Health and Social Care 2020[2, 3] |
| Emergency caesarean section with CC Score 4+, LOS 1 night | £3,302.05 | HRG Code NZ51A short stay. National Schedule of NHS Costs 2018-19 inflated using Curtis LA & Burns A. Unit Costs of Health and Social Care 2020[2, 3] |
| Emergency caesarean section with CC Score 0-1, LOS >1 and <=10 | £5,194.43 | HRG Code NZ51C non-elective long stay. National Schedule of NHS Costs 2018-19 inflated using Curtis LA & Burns A. Unit Costs of Health and Social Care 2020[2, 3] |
| Emergency caesarean section with CC Score 2-3, LOS >1 and <=11 | £5,980.42 | HRG Code NZ51B non-elective long stay. National Schedule of NHS Costs 2018-19 inflated using Curtis LA & Burns A. Unit Costs of Health and Social Care 2020[2, 3] |
| Emergency caesarean section with CC Score 4+, LOS >1 and <=11 | £7,227.73 | HRG Code NZ51A non-elective long stay. National Schedule of NHS Costs 2018-19 inflated using Curtis LA & Burns A. Unit Costs of Health and Social Care 2020[2, 3] |
| Planned caesarean section with CC Score 0-1, LOS 1 night | £2,915.21 | HRG Code NZ50C short stay. National Schedule of NHS Costs 2018-19 inflated using Curtis LA & Burns A. Unit Costs of Health and Social Care 2020[2, 3] |
| Planned caesarean section with CC Score 2-3, LOS 1 night | £2,957.69 | HRG Code NZ50B short stay. National Schedule of NHS Costs 2018-19 inflated using Curtis LA & Burns A. Unit Costs of Health and Social Care 2020[2, 3] |
| Planned caesarean section with CC Score 4+, LOS 1 night | £3,138.31 | HRG Code NZ50A short stay. National Schedule of NHS Costs 2018-19 inflated using Curtis LA & Burns A. Unit Costs of Health and Social Care 2020[2, 3] |
| Planned caesarean section with CC Score 0-1, LOS >1 and <=6 | £4,257.33 | HRG Code NZ50C non-elective long stay. National Schedule of NHS Costs 2018-19 inflated using Curtis LA & Burns A. Unit Costs of Health and Social Care 2020[2, 3] |
| Planned caesarean section with CC Score 2-3, LOS >1 and <=7 | £4,811.23 | HRG Code NZ50B non-elective long stay. National Schedule of NHS Costs 2018-19 inflated using Curtis LA & Burns A. Unit Costs of Health and Social Care 2020[2, 3] |
| Planned caesarean section with CC Score 4+, LOS >1 and <=10 | £5,790.91 | HRG Code NZ50A non-elective long stay. National Schedule of NHS Costs 2018-19 inflated using Curtis LA & Burns A. Unit Costs of Health and Social Care 2020[2, 3] |
| *Home delivery* |  |  |
| Spontaneous vaginal delivery, CC score 0, home birth | £1,552.93 | HRG Code NZ30C. National Schedule of NHS Costs 2018-19 inflated using Curtis LA & Burns A. Unit Costs of Health and Social Care 2020[2, 3] |
| *Terminations and Miscarriages* |  |  |
| Medical, Abortion or Miscarriage Care over 20 weeks Gestation, LOS 1 night | £1,567.11 | HRG Code MA53Z short stay. National Schedule of NHS Costs 2018-19 inflated using Curtis LA & Burns A. Unit Costs of Health and Social Care 2020[2, 3] |
| Medical, Abortion or Miscarriage Care from 14 to 20 weeks Gestation, LOS 1 night | £1,264.22 | HRG Code MA54Z short stay. National Schedule of NHS Costs 2018-19 inflated using Curtis LA & Burns A. Unit Costs of Health and Social Care 2020[2, 3] |
| Medical, Abortion or Miscarriage Care over 20 weeks Gestation, LOS >1 night | £4,406.55 | HRG Code MA53Z non-elective long stay. National Schedule of NHS Costs 2018-19 inflated using Curtis LA & Burns A. Unit Costs of Health and Social Care 2020[2, 3] |
| Medical, Abortion or Miscarriage Care from 14 to 20 weeks Gestation, LOS > 1 night | £3,488.77 | HRG Code MA54Z non-elective long stay. National Schedule of NHS Costs 2018-19 inflated using Curtis LA & Burns A. Unit Costs of Health and Social Care 2020[2, 3] |
| *Stillbirth* |  |  |
| Post-mortem and investigations and consultant follow-up visit to discuss findings | £1011.70 | Campbell et al. 2018 inflated using Curtis LA & Burns A. Unit Costs of Health and Social Care 2020[3, 5] |
| *Transfusion* |  |  |
| Unit of red blood cells | £133.44 | NHS Blood and Transplant Prices. https://nhsbtdbe.blob.core.windows.net/umbraco-assets-corp/16611/ncg-pricing-proposals-board-paper-19_62.pdf[6] |
| *Maternal transfer to another hospital* |  |  |
| Ambulance transfer | £260.34 | HRG Code ASS02. National Schedule of NHS Costs 2018-19 inflated using Curtis LA & Burns A. Unit Costs of Health and Social Care 2020[2, 3] |
| *Neonatal Unit (NNU)* |  |  |
| Baby inpatient day on NNU | £848.29 | Weighted average of HRG codes XA01Z, XA02Z, XA03Z and XA04Z (CCU13). National Schedule of NHS Costs 2018-19 inflated using Curtis LA & Burns A. Unit Costs of Health and Social Care 2020[2, 3] |
| *Neonatal ward* |  |  |
| Baby inpatient day on ward | £297.82 | HRG Code XA05Z (CCU15). National Schedule of NHS Costs 2018-19 inflated using Curtis LA & Burns A. Unit Costs of Health and Social Care 2020[2, 3] |
| *Baby transfers to another hospital* |  |  |
| Neonatal critical care transportation | £1273.34 | HRG Code XA06Z (CCU13). National Schedule of NHS Costs 2018-19 inflated using Curtis LA & Burns A. Unit Costs of Health and Social Care 2020[2, 3] |

MAU: Maternity Assessment Unit, LOS: length of stay, CC: comorbidities / complications

*Used to cost the MAU visits of individuals without an obstetric history of pre-eclampsia and without a diagnosis of hypertension.

†Used to cost the MAU visits of individuals with an obstetric history of pre-eclampsia and / or with a diagnosis of hypertension.

‡Information on the type of health professional seen was not available and so an average unit cost across midwifery and obstetric specialities was used when costing.

§In the absence of reasons for hospital admissions, inpatient stays were assumed to have been for monitoring of the individual and were costed using an excess bed-day cost.
